# *In vivo* Metabolism of Nifurtimox and the Drug-Drug Interaction Potential Including its Major Metabolites

**DOI:** 10.2174/1389200224666230817114758

**Published:** 2023-10-17

**Authors:** Simone I. Schulz, Dieter Lang, Gabriele Schmuck, Michael Gerisch, Michaela Bairlein, Robert Fricke, Heino Stass

**Affiliations:** 1 Bayer AG, 42096, Wuppertal, Germany

**Keywords:** Nifurtimox, biotransformation, metabolites, drug disposition, stability, drug-drug interactions, Chagas disease

## Abstract

**Background:**

Nifurtimox is an effective treatment for patients with Chagas disease, but knowledge of its biotransformation and excretion is limited.

**Objective:**

This study aimed to better understand the fate of oral nifurtimox *in vivo*.

**Methods:**

We investigated the exposure and excretion pathways of [^14^C]-labeled nifurtimox and its metabolites in rats. We then quantified the prominent metabolites and nifurtimox in the urine and plasma of patients receiving nifurtimox using LC-HRMS with reference standards and quantified these compounds in rat plasma after a single, high dose of nifurtimox. We also investigated potential drug-drug interactions (DDIs) of these compounds *in vitro*.

**Results:**

In rats, orally administered nifurtimox was rapidly absorbed (t_max_ 0.5 h) and eliminated (t_½_ 1.4 h). Metabolism of nifurtimox yielded six predominant metabolites (M-1 to M-6) in urine and plasma, and the dose was excreted equally *via* the renal and fecal routes with only traces of unchanged nifurtimox detectable due to its instability in excreta. In patients with Chagas disease, only M-6 and M-4 achieved relevant exposure levels, and the total amount of excreted metabolites in urine was higher in fed *versus* fasted patients, consistent with the higher systemic exposure. For nifurtimox, M-6, and M-4, no potential perpetrator pharmacokinetic DDIs with the main cytochrome P-450 enzymes and drug transporters were identified *in vitro*.

**Conclusion:**

This contemporary analysis of the complex metabolite profile and associated exposures emerging after oral dosing of nifurtimox in rats and humans, together with the expected low risk for clinically relevant DDIs, expands the understanding of this important anti-trypanosomal drug.

## 1 INTRODUCTION

Infection with the parasite *Trypanosoma cruzi* causes Chagas disease in humans, which remains an important cause of morbidity and premature death [1]. Globally, 6-7 million individuals are currently estimated to be infected with *T. cruzi* [2], and another 65-100 million people worldwide live in areas at risk for infection [3]. Nifurtimox is one of two approved treatments for Chagas disease [4] and was first trialed in adult patients in the 1960s [5, 6]. Although nifurtimox has been used for many years in several Latin American countries, it was only approved in the USA in 2020 for the treatment of pediatric patients with Chagas disease [7]. Contemporary investigations that supersede the largely historical studies [8-11] characterizing the pharmacokinetics (PK) of nifurtimox and its absorption, distribution, metabolism, and excretion (ADME) profile in humans and model mammalian systems have been completed only recently [12, 13]. From the perspective of current regulatory requirements, such preclinical and early clinical development studies are often incomplete, and a more detailed understanding of the behavior of nifurtimox in humans would be beneficial, particularly how it is metabolized, and the degree to which individuals taking nifurtimox are exposed to its metabolites.

Historical studies on animals have determined that the absorption and excretion of nifurtimox are rapid and complete [8, 9]. Studies on rats dosed orally with [^35^S]-labeled nifurtimox found that about half of the administered radioactivity was absorbed from the gastrointestinal tract within 4 h of dosing; a similar rate of absorption was also reported in dogs [8]. Within 2-3 days of oral or i.v. dosing in rats and dogs, 40-50% of the administered radioactivity was detected in urine, and much of the remaining activity was excreted in feces [8]. Thin-layer radiochromatographic analysis of urine collected from rats 24 h post-dose identified at least 10 radiolabeled excretion products and only small amounts of unchanged nifurtimox. A similar excretion profile was seen in dog urine [8]. Medenwald *et al*. quantified unlabeled nifurtimox excreted in urine from humans, dogs, and rats after oral administration and similarly detected no more than 0.5% of the administered dose in all cases [9]. The study by Medenwald *et al*. and a subsequent study with seven healthy volunteers determined that the time from oral administration of nifurtimox to the maximum plasma drug concentration (t_max_) was approximately 2-3 hours, with an average elimination half-life (t_½_) of approximately 3 hours [10]. This half-life was confirmed in a more recent Phase 1 food-effect PK study, in which t_max_ was estimated as 3 h in fasting patients and 4 h in those who had eaten before dosing [12]. An important finding of this food-effect study was that, for the same nifurtimox dose, exposure based on area under the plasma drug concentration curve (AUC) was 70% greater in fed compared to fasting patients [12].

**Fig. (1):**
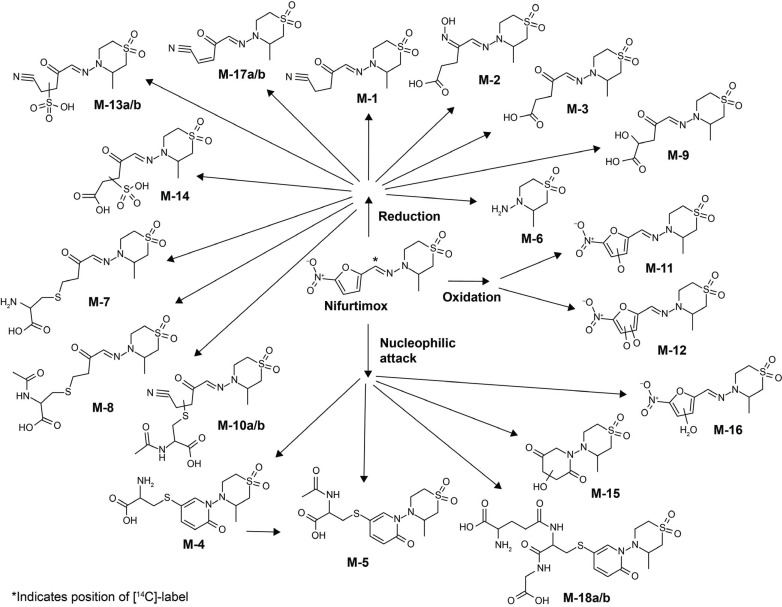
Nifurtimox and its metabolites [13]. Reprinted from Lang, D., Schulz, S.I., Piel, I., Tshitenge, D.T., Stass, H. Structural and mechanistic investigation of the unusual metabolism of nifurtimox. *Chem. Res. Toxicol*., **2022**, *35*, 2037-2048, © 2022, with permission from the American Chemical Society.

Although early studies demonstrated that the metabolism of nifurtimox is extensive, 50 years elapsed before a detailed characterization of the metabolites of nifurtimox was reported [13]. Analysis of urine from rats dosed orally with [^14^C]-nifurtimox identified at least 30 radiolabeled products, albeit most of them in trace amounts. Leveraging this information when scouting for unlabeled metabolites in urine from participants in a Phase 1 clinical trial of nifurtimox [12] helped to identify 18 products at levels sufficient to propose structures based on their high-resolution mass spectrometry (HRMS) fragmentation patterns [13]. Of these 18 metabolites, six (designated M-1 to M-6) were particularly abundant (Fig. **1**), and their proposed structures were confirmed by analysis (HRMS and two-dimensional nuclear magnetic resonance spectrometry) of reference standards synthesized based on the proposed structures. A key finding of this study was that nifurtimox metabolism does not appear to involve any classical hepatic or renal drug-metabolizing enzymes and is instead degraded by reductive and nucleophilic modifications, which are mediated at least in part non-enzymatically [13].

In the current study, we investigated the exposure and excretion pathways of nifurtimox and its metabolites in a mass balance study in rats using [^14^C]-labeled nifurtimox. We then quantified nifurtimox and its prominent metabolites in urine and plasma from patients with Chagas disease. Metabolites present at relevant systemic exposure levels, as well as nifurtimox, were assessed *in vitro* for possible PK drug-drug interactions (DDIs) of clinical significance. These metabolites were also quantified in a high-dose rat PK study for coverage in rats as the toxicological species.

## 2 MATERIALS AND METHODS

### 2.1 Materials

Radiolabeled substrate [^14^C]-nifurtimox [(*E*)-*N*-(3-methyl-1,1-dioxo-1,4-thiazinan-4-yl)-1-(5-nitrofuran-2-yl) methanimine)] with a specific activity of 6.40 MBq/mg was synthesized by American Radiolabeled Chemicals, Inc. (Saint Louis, MO, USA), incorporating ^14^C in the imine moiety. Unlabeled metabolites M-1 to M-5 were synthesized by Bayer AG (Wuppertal, Germany) for use as reference compounds in quantitative analyses, as previously reported [13]. M-6 is a synthetic precursor of nifurtimox, and was already available (Bayer AG, Wuppertal, Germany).

### 2.2 Investigation of the Disposition of [^14^C]-Nifurtimox and its Metabolites in Rats

The PK parameters of total radioactivity, nifurtimox, and metabolites, as well as the mass balance and metabolite profiles, were investigated in intact or bile duct-cannulated (BDC) male Wistar rats given a single oral or i.v. dose of [^14^C]-nifurtimox, respectively. After an adaption period, the animals were prepared for the study. Bile duct cannulation was performed on selected animals the day before their use in experiments.

In a mass balance study (n = 5) and a PK study (n = 2 per timepoint), 2.5 mg/kg b.w. [^14^C]-nifurtimox (3.61 MBq/mg) dissolved in 45% PEG 400, 10% ethanol, and 45% water (v/v/v) (0.5 mg/mL) was administered orally by gavage. For i.v. administration, 2.5 mg/kg b.w. [^14^C]-nifurtimox (3.61 MBq/mg) dissolved in 45% PEG 400, 10% ethanol, and 45% water (v/v/v) (0.5 mg/mL) was injected as a single i.v. bolus directly into the bloodstream *via* a lateral tail vein over a period of up to 30 s (n = 5). In an additional stability study, male Wistar rats (n = 3 per timepoint) were administered 2.5 mg/kg b.w. [^14^C]-nifurtimox (3.58 MBq/mg) dissolved in 45% PEG 400, 10% ethanol, and 45% water (v/v/v) (0.5 mg/mL) orally. The total radioactivity concentration in the dosage form was confirmed by liquid scintillation counting (LSC).

#### 2.2.1 Sample Collection and Processing

Animals were housed in suitable metabolism cages. Blood was collected from separate rats at 0.25, 0.5, 1, 1.5, 2, 3, 5, and 8 h post-dose and stored in lithium heparinized tubes. Blood cells and plasma were separated by centrifugation. Plasma was stored at ≤-15°C prior to analysis. Urine and feces from intact rats were collected at specified time intervals up to 120 h. Urine, bile, and feces from BDC rats were collected at specified time intervals up to 24 h. At the end of the study, the cages were rinsed with water, and samples were collected. All biological samples were weighed immediately. Feces samples were homogenized with water to obtain a uniform radioactivity distribution. All samples were stored deep-frozen at ≤-15/-70°C before further processing. During the necropsy, selected organs and tissues were collected and wet-weighed. The remaining carcass was also wet-weighed. The carcass and all tissues were homogenized and stored at room temperature prior to processing for analysis. In the stability study, blood and urine were collected at 0.25 and 0.5 h post-dose. Plasma and urine were immediately frozen in liquid nitrogen and stored at -80°C to prevent degradation. The total radioactivity in samples and tissues was determined by LSC after appropriate sample preparation.

#### 2.2.2 Biotransformation of [^14^C]-Nifurtimox in Rats

Extraction recovery of nifurtimox-related radioactivity after work-up for rat plasma and feces and as control for rat urine and bile was determined by LSC on a Tri-Carb® 2910 and 4910 (Perkin Elmer LAS GmbH, Rodgau, Germany) with automatic quench correction by the external standard channel ratio method at 13°C using Ultima Gold^™^ high flash point scintillation cocktail (Perkin Elmer LAS GmbH, Rodgau, Germany).

Aliquots of individual plasma samples were pooled separately for each timepoint by equal volume. For protein precipitation, pooled plasma was treated with a three-fold volume of acetonitrile. Samples were homogenized and precipitated by centrifugation (20,000 *g*, 5 min). Samples were concentrated and analyzed using high-performance liquid chromatography (HPLC) with off-line radioactivity detection by LSC (HPLC-LSC). Extraction recoveries of radiolabeled nifurtimox-associated analytes after the work-up of pooled plasma samples were between 66.8-88.4% (PK study) and 67.5-104.7% (stability study).

Aliquots of individual urine or bile samples were pooled separately for each time interval by equal volume and analyzed using HPLC-LSC. Aliquots of individual feces samples were pooled separately for each time interval, ensuring that each aliquot contained the same amount of radioactivity. The pooled samples were extracted twice with a mixture of acetonitrile/water (80:20 v/v, 2 x 0.5 mL). The resulting suspensions were centrifuged (20,000 *g*, 5 min), and the supernatants were then combined for each time interval, concentrated, and subjected to HPLC-LSC. The mean extraction recovery of radiolabeled nifurtimox-associated analytes after the work-up of pooled feces samples was 87.5% for intact rats and 67.6% for BDC rats.

#### 2.2.3 Data Evaluation and Pharmacokinetic Analysis

The PK parameters were calculated by noncompartmental analysis based on the mean plasma concentrations of total radioactivity, nifurtimox, and its metabolites using ToxKin (Entimo, Berlin, Germany). From the concentration-time data, the maximum plasma concentration (C_max_), time to C_max_ (t_max_), area under the plasma concentration-time curve from time 0 h to 8 h (AUC_(0-8)_), relative AUC_(0-8)_, AUC from time 0 h extrapolated to infinity (AUC_(0-∞)_), and terminal half-life (t_½_) were calculated.

The amount of total radioactivity in urine, feces, bile, and tissues was determined by multiplying the volume or weight of the samples by the respective total radioactivity concentration. The amount of radioactivity recovered in the excreta (A_E_), cage wash, and tissues was compared with the administered radioactive dose to establish the radioactive balance. Cumulative excretion was calculated as the sum of the excretion for each collection interval. Arithmetic means and coefficients of variation (CV) were calculated for these percentages. During metabolite profiling, the concentration or amount of each analyte for each sample and timepoint or interval was calculated by multiplying the total radioactivity concentration or amount excreted of each sample with the percent area of each analyte obtained after HPLC-LSC separation.

#### 2.2.4 Stability of [^14^C]-Nifurtimox in Rat Excreta

For the investigation of nifurtimox stability in urine and to allow comparison of the metabolite profiles in urine and plasma, early post-dose timepoints were investigated (Section 2.2.1). One aliquot of a pooled urine sample taken at 0.25 h post-dose was analyzed immediately using HPLC-LSC. Further aliquots were analyzed after storage for 24 h at room temperature. To assess the stability of nifurtimox in feces, [^14^C]-nifurtimox (8.8 µM) was incubated with a suspension of fresh rat feces (approximately 250 mg) in degassed phosphate buffer pH 7.0/deionized water (1:5, v/v) under anaerobic conditions (argon atmosphere). After incubation for 22.5 h at 37°C with shaking, incubations were terminated by the addition of acetonitrile, then extracted (three times with 2 mL acetonitrile for 30 min) and centrifuged (1726 *g*, 3 min). The combined supernatant was analyzed directly using HPLC-LSC.

#### 2.2.5 Analytical HPLC-LSC

Metabolite profiles were analyzed by HPLC-LSC using either a 1290 Infinity or 1260 LC system (Agilent Technologies, Waldbronn, Germany), Ultima Flo^™^ AP scintillation cocktail (Perkin Elmer LAS GmbH, Rodgau, Germany), and a Perkin Elmer 1450 and 2450 Microbeta2^TM^ Plus Liquid Scintillation Counter (Turku, Finland) with a 5 s sampling time. Gradient elution was performed at 0.3 mL/min and 40°C with a Prodigy ODS-3 reversed-phase column (150 x 2 mm, particle size 3 μm; pore size 100 Å) (Phenomenex, Aschaffenburg, Germany) with a mobile phase of A: 0.05% formic acid (0.1% for *ex vivo* feces incubation) and B: acetonitrile + 0.05% formic acid (0.1% for *ex vivo* feces incubation). The gradient in the mobile phase was: 0 min, 0% B; 4 min, 0% B; 26 min, 37% B; 27 min, 90% B; 29 min, 90% B; 31 min, 0% B; 32 min, 0% B. For all samples, the recovery of eluted radioactivity was verified by comparison of the amount of radioactivity initially injected and the integrated HPLC area per sample.

### 2.3 Quantification of Nifurtimox and its Metabolites in Rat Plasma

Nifurtimox and metabolites M-1 to M-6 were quantified in rat plasma samples from the male (n = 4) and female (n = 4) Wistar rats taken after oral administration of a single 200 mg/kg dose of nifurtimox (in 1% hydroxypropylmethyl cellulose in water, m/v). Blood samples taken from individual animals at each timepoint (0.25, 0.5, 1, 1.5, 2, 3, 5, 8, 24, 32, 48, 56, and 72 h post-dose) were collected into lithium heparinized tubes. Blood cells and plasma were then separated by centrifugation, and the plasma was stored at ≤-15°C until required. For analysis, plasma samples were thawed at room temperature and homogenized by vortex mixing. Plasma samples were pooled separately for male and female rats by combining 25 µL from each of the 4 animals for each timepoint. Calibration standards and quality control samples of nifurtimox and metabolites M-1 to M-6 were prepared by diluting stock solutions in plasma from untreated animals. To each pooled plasma (100 µL), calibration, or control sample, 300 µL of an internal standard solution containing [^2^H_8_]-nifurtimox (500 ng/mL) and [^2^H_3_]-M-6 (2500 ng/mL) in acetonitrile + 0.5% formic acid in water (v/v) was added and mixed thoroughly. All samples were then centrifuged (20,000 *g*, 5 min) to precipitate the protein. The supernatant was analyzed using a 1290 Infinity LC system equipped with a diode array detector and an Orbitrap Q-Exactive Plus HRMS (Thermo Fisher Scientific, Bremen, Germany) with a heated electrospray ionization source. HPLC was performed at 0.3 mL/min and 40°C using a Synergi Polar-RP reversed-phase column (150 × 2 mm, particle size 4 µm; pore size, 80 Å) (Phenomenex, Aschaffenburg, Germany) with a mobile phase of A: 0.05% formic acid and B: acetonitrile + 0.05% formic acid. The mobile phase gradient was: 0 min, 0% B; 2.5 min, 0% B; 3 min, 20% B; 8 min, 45% B; 9 min, 90% B; 10.1 min, 90% B; 12 min, 0% B; 13 min, 0% B. The following HRMS parameters were used: mass range, 120-200 Da (0-2.5 min) and 230-600 Da (2.5-13 min) in full scan mode; resolution, 280,000; ion source voltage, 3500 V; capillary temperature, 300°C; source temperature, 60°C.

The calibration was based on the relative peak area as a function of concentration obtained by linear regression. Quantification using Xcalibur^™^ Quan Browser software (Thermo Fisher Scientific, USA) was performed on a mass basis [μg/mL], but the final calculations for the exposure in plasma were done on a molar basis because of the substantial differences (up to ~2.5-fold) of the molecular masses of nifurtimox and metabolites M-1 to M-6. Thus, all concentrations in μg/mL were back-calculated to nmol/mL. The PK parameters for each analyte were calculated using ToxKin software, as described above.

### 2.4 Quantification of Nifurtimox and its Metabolites in Human Urine

Quantification of nifurtimox and metabolites M-1 to M-6 was performed in human urine samples from patients with chronic Chagas disease after the oral intake of 120 mg single-dose nifurtimox [12] using a novel, partially validated method developed for this investigation. Urine samples were collected from fasted and fed patients pre-dose (0 h) and at four post-dose intervals (0-4, 4-8, 8-12, and 12-24 h) and stored frozen until required. For analysis, urine samples were thawed at room temperature and homogenized by vortex mixing. Calibration standards and quality control samples of nifurtimox and metabolites M-1 to M-6 were prepared by the dilution of stock solutions in urine from a healthy volunteer. Equal volumes (75 µL) of an internal standard solution of [^2^H_8_]-nifurtimox dissolved in water (10 µg/mL) were added to each urine, calibration, or control sample and mixed thoroughly. Samples from 6 patients were then analyzed using liquid chromatography-high-resolution mass spectrometry (LC-HRMS) with a 1290 Infinity LC system equipped with a diode array detector and an Orbitrap Fusion^™^ Lumos^™^ Tribrid^™^ HRMS (Thermo Fisher Scientific, Bremen, Germany) with a heated electrospray ionization source. For quantification, the LC conditions were as described above for analysis of rat plasma, and the HRMS parameters were: mass range, 120-600 Da in full scan mode and all ion fragmentation in positive ionization mode; resolution, 240,000; ion source voltage, 3500 V; capillary temperature, 300°C; source temperature, 60°C.

The system was calibrated using the relative peak area as a function of concentration, which was obtained by linear regression. Quantification was performed on a mass basis (μg/mL), with the final calculation for the percentage of dose excreted in urine made on a molar basis to account for the substantial differences (up to ~2.5-fold) in the molecular masses of the compounds of interest. The analytical precision for all analytes was in the range of 1.1% to 4.4%, with an accuracy of 94% to 107%. All concentrations in μg/mL were back-calculated to nmol/mL, and these concentrations were then used to calculate a total molar amount based on the urine volume collected during each interval. The dose of nifurtimox administered (120 mg) was calculated as 418 µmol.

### 2.5 Quantification of Nifurtimox and its Metabolites in Human Plasma

Quantification of nifurtimox and metabolites M-1 to M-6 was performed in plasma samples from patients with chronic Chagas disease taken after oral administration of 120 mg single-dose nifurtimox under fed conditions [14] using a similar partially validated method to that described above (Section 2.4). Plasma samples from timepoints 0, 0.25, 0.5, 0.75, 1, 1.5, 2, 2.5, 3, 4, 6, 8, 12, 15, and 24 h from 6 patients were analyzed. Blood samples were collected into lithium heparinized tubes. Blood cells and plasma were then separated by centrifugation, and the plasma was stored frozen until needed. For the analysis, plasma samples were thawed at room temperature and homogenized by vortex mixing. Equal volumes of plasma from the six patients for each timepoint were combined to a pooled volume of 200 µL. To precipitate protein, a 3-fold volume of acetonitrile/0.5% formic acid (v/v in water) was added to each pooled sample which was thoroughly mixed and then centrifuged at 20,000 *g*. The supernatant (about 720 μL) was concentrated to 100-120 μL to remove excess acetonitrile and analyzed directly by LC-HRMS using the same system described above for the analysis of rat plasma, but with the following gradient in the mobile phase: 0 min, 0% B; 1.5 min, 0% B; 3 min, 30% B; 8 min, 45% B; 9 min, 90% B; 10.1 min, 90% B; 12 min, 0% B; 13 min, 0% B. The following HRMS parameters were used: mass range, 120-600 Da (0-2 min) and 230-600 Da (2-13 min) in full scan mode; resolution, 280,000; ion source voltage, 3500 V; capillary temperature, 300°C; source temperature, 60°C. For all analytes, the analytical precision ranged from 0.1% to 8.8%, and the accuracy was 95% to 108%. The concentrations of nifurtimox and metabolites M-1 to M-6 in the plasma samples were calculated in the same way that rat plasma concentrations were calculated.

### 2.6 *In vitro* Investigations of Potential Drug-drug Interactions of Nifurtimox, M-6, and M-4

*In vitro* studies addressing the drug-drug interaction (DDI) potential were conducted on nifurtimox and metabolites M-6 and M-4. The inhibition of cytochrome P450 (CYP) isoforms and a range of cellular transporters (except multidrug and toxin extrusion [MATE] proteins), as well as the induction of CYP isoforms were investigated using previously described methods [15, 16]. *In vitro* studies to investigate the inhibitory potential of nifurtimox, M-6, and M-4 for MATE1 and MATE2K are described in the Supplementary Material.

## 3 RESULTS

### 3.1 Absorption, Excretion, and Recovery of Total Radioactivity in Rats

**Fig. (2):**
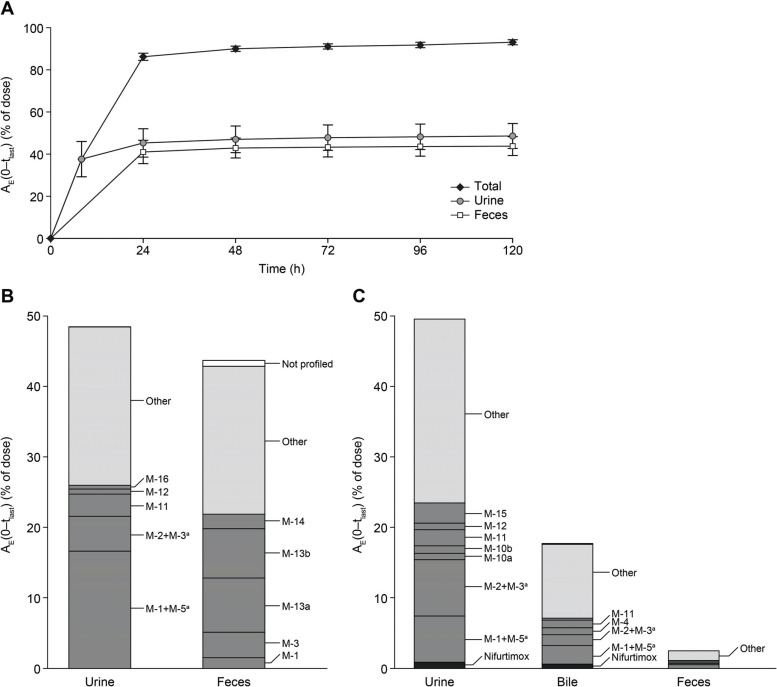
Cumulative excretion of total radioactivity (**A**); excreted components as a percentage of dose in urine and feces samples following oral administration of [^14^C]-nifurtimox to intact male Wistar rats (**B**); and in urine, feces, and bile samples following i.v. administration of [^14^C]-nifurtimox to male BDC Wistar rats (**C**). In **B** and **C**, excreted components >0.5% of the dose are labeled. ^a^M-1+M-5 and M-2+M-3 were analyzed as pairs of metabolites because separation could not be achieved chromatographically.

The absorption of radioactivity in male Wistar rats (n = 5) was rapid after oral dosing with 2.5 mg/kg [^14^C]-nifurtimox (t_max_, 0.5 h; C_max_, 1010 μg-eq/L). Most of the administered radioactivity (approximately 86%) was excreted within 24 h, and almost all radioactivity (92.4% of dose) was excreted within 120 h (Fig. **2A**). Approximately equal amounts of radioactivity were excreted in urine (48.6% of dose) and feces (43.8% of dose). Total recovery, including excreta, residues, and cage wash at 120 h after oral dosing, was 97.3% (CV 1.25%). Following i.v. administration of 2.5 mg/kg [^14^C]-nifurtimox to BDC rats (n = 5), 49.6% and 17.7% of the administered dose were excreted in urine and bile, respectively, within 24 h. In addition, 6.9% of the administered dose was secreted directly into the gut (4.5% in the gastrointestinal tract, 2.5% in feces). Recovery at 24 h after i.v. administration to BDC rats was 94.2% (CV 2.0%) of the administered radioactivity, including 17.6% in residues.

### 3.2 PK of Total Radioactivity, [^14^C]-Nifurtimox, and its Metabolites in Rats

**Fig. (3):**
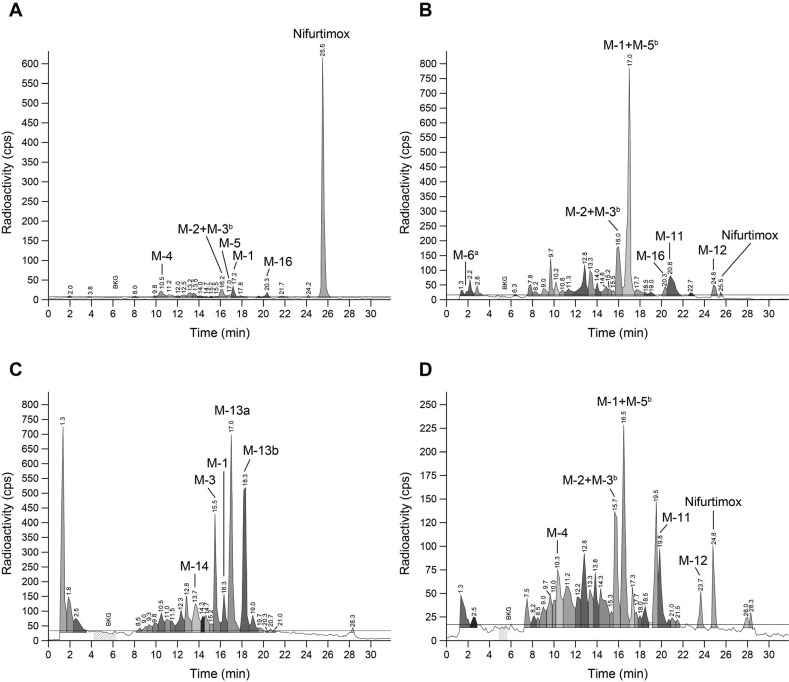
Representative HPLC chromatograms of [^14^C]-nifurtimox and metabolites after oral dosing of male Wistar rats in pooled plasma collected at 0.5 h post-dose (**A**), in pooled urine collected 0-8 h post-dose (**B**), in pooled feces collected 0-24 h post-dose (**C**); and also in pooled bile collected 0-8 h after i.v. dosing of male BDC Wistar rats (**D**). cps, counts per second; ^a^Unlabeled metabolite M-6 detected by HRMS; ^b^M-1+M-5 and M-2+M-3 were analyzed as pairs of metabolites because separation could not be achieved chromatographically.

Metabolite profiling using HPLC-LSC in pooled plasma from male Wistar rats dosed orally with 2.5 mg/kg [^14^C]-nifurtimox revealed nifurtimox and the metabolites M-1, M-2, M-3, M-4, M-5, and M-16 (Fig. **3A**). M-6 did not contain the [^14^C]-label and was only qualitatively identified by LC-HRMS. PK parameters determined for total radioactivity, nifurtimox, and metabolites M-1 to M-5 are summarized in Table **1**. Nifurtimox was the predominant component, representing 37.9% of total radioactivity AUC_(0-8)_. Among the metabolites, M-1 (a saturated open-chain nitrile) showed the highest exposure with 16.1% of total radioactivity AUC_(0-8)_. Further minor circulating metabolites were M-2+M-3, M-4, and M-5, each showing exposures in the range of 2-3% of total radioactivity AUC_(0-8)_. The remaining radioactivity comprised multiple minor unidentified drug-related components. The plasma t_½_ for total radioactivity was 2.9 h compared to 1.4 h for nifurtimox in the interval up to 8 h.

### 3.3 Metabolite Profiling in Rat Excreta

In urine (48.6% of the administered dose), a very complex pattern of radiolabeled products emerged (Fig. **3B**) and about half of the radioactivity detected (53.6%) could be assigned to already identified metabolite structures (M-1, M-2, M-3, M-5, M-11, M-12, M-16) (Fig. **1**). Besides trace amounts of [^14^C]-nifurtimox (0.1% of administered dose), the most abundant products were M-1+M-5 (totaling 16.6% of the dose) and M-2+M-3 (totaling 5.0% of dose). Although unlabeled, M-6 was also detected by HRMS (Fig. **3B**) but not quantified. The remaining drug-related radioactivity (22.5% of dose) consisted of many (>10) minor unidentified products. Analysis of urine from BDC rats after i.v. administration of [^14^C]-nifurtimox also revealed only trace amounts of nifurtimox and identified the pairs of metabolites M-1+M-5 and M-2+M-3 as the most abundant (Fig. **2C** and Table **S1**).

As with urine, the pattern of metabolites in feces (43.8% of the administered dose) was complex, and half of the radioactivity (50.1%) in the feces could be assigned to identified structures (Fig. **3C**). No unchanged drug was detected. Two stereoisomeric open-chain sulfone metabolites, M-13a and M-13b, were the most abundant metabolites (7.7% and 7.0% of the dose, respectively). M-3 accounted for 3.6% of the administered dose and M-1 for 1.5%. The remaining drug-related radioactivity (21.0% of the dose) was comprised of at least 10 different products. The same metabolites were detected in the feces of BDC rats after i.v. administration (Fig. **2C** and Table **S1**).

Analysis of bile from BDC rats after i.v. administration (17.7% of the administered dose) also revealed a complex pattern of metabolites, and about 40% of the radioactivity could be assigned to identified structures (nifurtimox, M-1, M-2, M-3, M-4, M-5, M-11, and M-12) (Fig. **3D**). Besides trace amounts of nifurtimox (0.6% of dose), M-1+M-5 (2.7% of dose) and M-2+M-3 (1.6% of dose) represented the main components. The remaining drug-related radioactivity (10.5% of the dose) was comprised of at least 15 different products. The quantitative mass balance data, as a percentage of the administered dose up to 120 h after a single oral administration of 2.5 mg/kg [^14^C]-nifurtimox to male Wistar rats and up to 24 h after a single i.v. administration of 2.5 mg/kg [^14^C]-nifurtimox to male BDC Wistar rats, are shown in Figs. (**2B** and **2C**) and Table **S1**.

### 3.4 Stability of [^14^C]-Nifurtimox in Plasma, Urine, and Feces of Rats

**Fig. (4):**
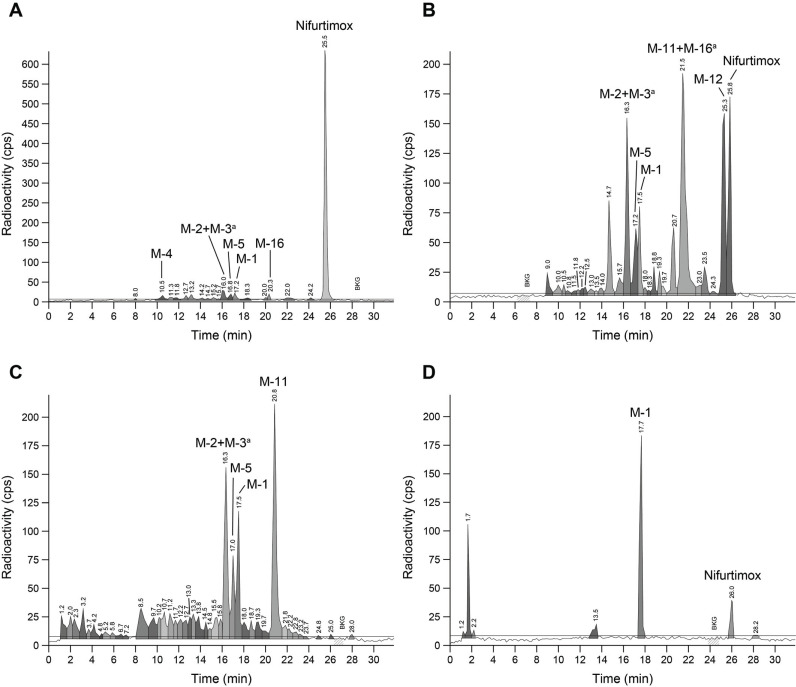
HPLC chromatograms of [^14^C]-nifurtimox and metabolites after oral dosing of male Wistar rats in pooled plasma collected at 0.25 h post-dose (**A**), in pooled urine at 0.25 h post-dose and analyzed immediately after sampling (**B**), in pooled urine at 0.25 h post-dose and analyzed following storage for 24 h at room temperature after sampling (**C**); and also in rat feces incubated with [^14^C]-nifurtimox (8.8 µM) at 37°C for 22.5 h under anaerobic conditions (**D**). cps, counts per second; ^a^M-2+M-3 and M-11+M-16 were analyzed as pairs of metabolites because separation could not be achieved chromatographically.

As the complex metabolite profiles in rat urine (*e.g*., the 0-8 h interval) (Fig. **3B**) were different from those seen in plasma up to 8 h post-dose (*e.g*., the 0.5 h profile) (Fig. **3A**), plasma and urine were collected for comparison at early timepoints from a different group of male Wistar rats after oral dosing with 2.5 mg/kg [^14^C]-nifurtimox. Each sample was immediately snap-frozen after collection to avoid the potential degradation of nifurtimox. At both 0.25 h (Fig. **4A**) and 0.5 h (not shown), post-dose nifurtimox was the main radiolabeled component in plasma (81.5% and 72.5% of sample radioactivity, respectively), with only trace amounts of metabolites, *e.g*., M-1 (0.25 h, 1.4%; 0.5 h, 2.3%). At 0.25 h post-dose, relevant quantities of unchanged nifurtimox (11% of sample radioactivity) were identified in rat urine (Fig. **4B**); this quantity decreased by approximately two-thirds by 0.5 h (4% of sample radioactivity). The persistence of nifurtimox as the dominant component in plasma, combined with the relatively rapid decrease in its levels in urine over time and the overall differences between the metabolite profiles of urine at up to 0.5 h post-dose and from 0-8 h post-dose (nifurtimox representing 0.3% of sample radioactivity, Fig. **3B**), suggested that nifurtimox was fundamentally unstable in rat urine.

The stability of nifurtimox was, therefore, further investigated by reanalyzing a pooled 0.25 h post-dose urine sample containing relevant amounts of [^14^C]-nifurtimox after storage at room temperature for 24 h (Fig. **4C**). Comparison with the metabolite profile immediately after sampling (Fig. **4B**) showed that nifurtimox was undetectable and metabolite levels (*e.g*., M-1) had concomitantly increased. It has previously been reported that nifurtimox is unstable in human excreta [13], and as described above, no nifurtimox was found in rat feces samples. Therefore, the stability of [^14^C]-nifurtimox in rat feces was determined. *Ex vivo* incubation of [^14^C]-nifurtimox (8.8 µM) in an aqueous suspension of fresh rat feces at 37°C for 22.5 h under anaerobic conditions yielded significant quantities of M-1 (Fig. **4D**), whereas no degradation of nifurtimox was observed in an aqueous control incubated without feces (data not shown), indicating that nifurtimox was unstable in rat feces.

### 3.5 Quantification of Nifurtimox and its Metabolites in Human Urine

**Fig. (5):**
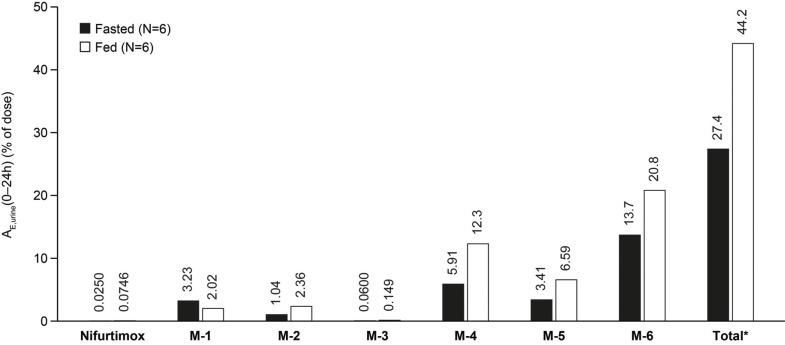
Mean amount excreted (A_E,urine_) in the percentage of an administered dose of nifurtimox and metabolites M-1 to M-6 in human urine from 6 fed and 6 fasted patients following a single 120 mg dose of nifurtimox. *Total = sum of amounts of nifurtimox and M-1 to M-6 excreted in urine within 24 h after a single oral dose.

Urine samples were collected from adults with Chagas disease while they were participating in a Phase 1 PK study that investigated food effects on plasma nifurtimox exposure [12]. Urine samples from six fed and six fasted patients were used for LC-HRMS quantification of nifurtimox and its most prominent metabolites, M-1 to M-6. Unchanged nifurtimox constituted, on average, less than 0.1% of the administered dose in urine (fed 0.07%; fasted 0.03%). Urinary metabolites were abundant in samples from both fed and fasted patients and are listed in this study in descending order based on the proportion of the administered dose in fed and fasted participants, respectively: M-6 (20.8% and 13.7%), M-4 (12.3% and 5.9%), M-5 (6.6% and 3.4%), M-1 (2.0% and 3.2%), M-2 (2.4% and 1.0%), and M-3 (0.15% and 0.06%) (Fig. **5**). In addition to the six quantified metabolites, many nifurtimox-related components were observed (oxidation or reduction products M-7 to M-12 and the glutathione conjugate M-18a/b) [13], revealing a similarly complex metabolite profile covering the same metabolites as seen in rat urine in the mass balance study. In total, an average of 44.2% (range, 37.0-55.9%) of the administered dose was recovered in urine from fed participants, which was attributable to nifurtimox and its six most prominent metabolites, compared with an average of 27.4% (range, 19.6-40.7%) recovered from fasting participants.

### 3.6 Quantification of Nifurtimox and its Metabolites in Human Plasma

Plasma samples were collected and pooled from six adult patients with Chagas disease participating in a phase 1 PK study [14]. Similarly to the urine analysis, nifurtimox and metabolites M-1 to M-6 were quantified in pooled plasma samples. Plasma PK parameters for nifurtimox and M-1 to M-6 are summarized in Table **2**, and the plasma concentrations of nifurtimox and the six metabolites over time are shown in Fig. (**S1**). The total quantified exposure was calculated as the sum of each AUC (for nifurtimox and the six metabolites), and the relative exposure of each component was expressed as a percentage of this total value. Apart from nifurtimox, which accounted for 16.9% of the total quantified exposure, M-6 (69.4%) and M-4 (8.9%) were the only metabolites among those quantified that showed relevant plasma exposure in humans and exceeded or approached the guideline threshold (10% of total exposure) [17, 18] for further safety and drug-drug interaction testing [19]. As was seen during the analysis of human urine, a complex pattern of at least 15 metabolites (including M-1 to M-6) was observed in plasma.

### 3.7 Quantification of Nifurtimox and its Metabolites in Rat Plasma after a Single, High Dose of Nifurtimox

Based on the exposure of nifurtimox metabolites in human plasma, the exposure of these metabolites in rats as the preclinical species for toxicological investigations was determined in a similar manner. Nifurtimox and M-1 to M-6 were quantified in pooled rat plasma from four male and four female Wistar rats after administration of a single, oral 200 mg/kg b.w. dose of nifurtimox, and plasma PK parameters were determined (Table **S2**). For metabolites M-6 and M-4 that exhibited relevant human plasma exposure, the exposures in rat plasma were at least 2-fold greater (M-6, 2.4-2.8-fold; M-4, 2.0-2.1-fold ratio of rat:human AUC_(0-24)_). These results confirm the suitability of rats as a toxicological species with regard to nifurtimox metabolism.

### 3.8 *In vitro* Investigation of Nifurtimox, M-6, and M-4 for Potential PK Drug-Drug Interactions

The perpetrator PK drug-drug interaction potential of nifurtimox and the most relevant human metabolites M-6 and M-4 towards human CYP enzymes and drug transporters were investigated for reversible and irreversible CYP inhibition, CYP induction, and the inhibition of efflux and uptake transporters. These three compounds showed no inhibitory or inductive effects on the CYP isoforms tested. For nifurtimox, M-6, and M-4, IC_50_ values for CYP inhibition were >50 µM, >100 µM, and >20 µM, respectively, and induction of CYP mRNA or activity was absent at up to 30 mg/L, 90 mg/L, and 80 mg/L, respectively (Table **S3**). Similarly, nifurtimox, M-6, and M-4 showed no or only marginal inhibition of a range of transporters (P-glycoprotein [P-gp], breast cancer resistance protein [BCRP], MATE1, MATE2K, organic anion transporting polypeptides [OATP] 1B1 and 1B3, organic anion transporters [OAT] 1 and 3, and organic cation transporter [OCT]) 2 (Table **S4**). Based on these *in vitro* data and considering unbound maximum plasma concentrations (Table **2**), no clinically relevant drug interactions or impact on CYP activities are expected at the therapeutic dose of nifurtimox.

## 4 DISCUSSION

We investigated the absorption, metabolism, and excretion of [^14^C]-labeled nifurtimox in male Wistar rats and quantified nifurtimox and metabolites M-1 to M-6 in plasma and urine from patients with Chagas disease receiving 120 mg unlabeled nifurtimox. Furthermore, nifurtimox and two metabolites with relevant systemic exposure in patients (M-6 and M-4) were investigated with respect to potential DDIs arising from the inhibition or induction of CYP isoforms or the inhibition of transporters, and these compounds were quantified in a high-dose rat PK study providing toxicological coverage of human exposure. Absorption of nifurtimox from the gut occurred rapidly in rats. In humans, absorption and exposure were substantially increased in fed patients compared to fasted patients, as previously reported [12]. Analysis of orally administered [^14^C]-nifurtimox in rats revealed that total drug-related material (the parent compound and radiolabeled metabolites) was eliminated in similar proportions *via* the renal (44%) and biliary/fecal (49%) route, indicating that both excretion routes are of similar importance. Similar proportions of excreted radiolabeled material in urine and feces after oral nifurtimox administration have been reported previously [8]. Modest amounts of radioactivity (<7% of dose) reached the gut within 24 h following i.v. nifurtimox administration to BDC rats, suggesting that direct secretion of nifurtimox and metabolites into the gut is not a significant elimination route. However, the quantification of unchanged nifurtimox in feces was compromised, as *ex vivo* incubations of feces showed the formation of relevant amounts of M-1, which could be attributed to the reduction of nifurtimox by gut microflora. Therefore, the hepatobiliary and/or direct secretion of unchanged nifurtimox into the gut cannot be excluded. In addition, the presence of relevant amounts of M13a/b and M-14 exclusively in feces suggests that sulfonation of appropriate precursors took place in the rat gastrointestinal tract. As the degradation of nifurtimox in the gut cannot be prevented, quantitative mass balance measurements in fecal samples probably do not represent the actual pattern of absorption and elimination of nifurtimox, and thus need careful interpretation.

Quantification and mass balancing of nifurtimox and metabolites M-1 to M-6 in urine from fed patients revealed that 44.2% of the administered dose was renally excreted, an amount very similar to that excreted in rat urine (48.6% of the dose). This suggests that there are no significant species differences in the excretion pathways. Although data for fecal excretion in humans are still needed, both the urinary and fecal routes of excretion would thus also be expected to be relevant in humans. Also, no qualitative species differences were observed between rats and humans regarding the metabolism of nifurtimox. Consistent with this finding is the observation that nifurtimox biotransformation is not mediated by common drug-metabolizing enzymes in the liver and kidneys, which could manifest species differences in the metabolic profiles of substrates of these enzymes [13]. Although the metabolic pathways are similar, some of the resultant metabolites were excreted differently in the two species. The systemic exposure of M-4, for example, is similar for both species, yet substantial amounts of M-4 were excreted in human urine while no M-4 was detected in rat urine. It has been recently shown [13] that M-4 is acetylated to M-5 in the kidneys, a process that seems to be more efficient in rats than humans, which could explain the difference in M-4 excretion between the two species.

Nifurtimox appears to be stable in plasma, but little is found in urine (rat or human), and the metabolite profile in urine is complex. In rats, assuming an oral bioavailability of 90% and an unbound fraction (f_u_) in plasma of 62.1%, the theoretical excretion based on glomerular filtration rate (GFR) corresponds to 23% of the dose, which contrasts with the observed urinary excretion of <1% of the dose. Similarly, approximately 10% of the nifurtimox dose would be expected to be excreted unchanged in human urine based on estimations accounting for GFR, f_u_ (57.6%), and AUC. The reported observation that nifurtimox exposure is increased in patients with chronic kidney disease compared with healthy individuals [20] is consistent with the assumption that there is renal clearance of unchanged nifurtimox. However, this data is limited to only seven patients for whom feeding status was undisclosed; as described in the present study and elsewhere, the timing of food intake relative to oral dosing of nifurtimox markedly affects subsequent exposure. In rat urine collected shortly after nifurtimox administration (0.25 h post-dose), relevant quantities of unchanged nifurtimox (11% of total sample radioactivity) were identified. Reanalysis of this urine revealed time-dependent degradation of nifurtimox, and only low levels of the compound were detected in samples collected during longer intervals after dosing (0-8 h), suggesting that there is renal clearance of unchanged nifurtimox. Overall, the findings from the analyses of rat urine immediately after sampling and the stability studies, together with estimates based on the GFR and unbound drug fraction, suggest that a relevant proportion of nifurtimox was excreted unchanged in the urine. The conclusion that there is relevant renal clearance of nifurtimox differs from previous interpretations suggesting that low levels of nifurtimox in urine are the result of the almost complete metabolism of the drug in the body.

The metabolite profiles of nifurtimox in rats indicate that there are at least 30 metabolites, of which approximately half could be structurally identified, as reported here and in previous studies [13]. The most common nifurtimox metabolites in plasma were M-1 to M-6 and M-16. All other metabolites were detected only in trace amounts, and no metabolites unique to humans were identified. Reduction of nifurtimox yields M-1, M-2, M-3, and M-6, while M-4 and, indirectly, M-5 result from nucleophilic attack by cysteine and rearrangement [13]. Quantification of M-1 to M-6 (calibrated against synthetic references) in human plasma determined that M-6 is the only metabolite to which humans are significantly exposed. However, exposure to M-4 approached a level high enough to merit further investigation. Similarly, quantification of M-1 to M-6 in human urine confirmed M-6 and M-4 as the two most abundant metabolites among those that were quantified. Further quantification of metabolites M-1 to M-6 in plasma from rats following a single high dose of nifurtimox found that the exposure levels of nifurtimox, M-6, and M-4 were greater in rats than in humans, demonstrating that the exposure of rats to the two metabolites of most clinical relevance (M-6 and M-4) is sufficient to justify the use of this species for toxicological assessments.

The pharmacological activity of M-6 and M-4 was not expected, as neither compound possesses a nitro group, which is considered to be essential [21]. In addition to nifurtimox, M-6 and M-4 were identified as plasma metabolites with relevant exposure, meriting the investigation of their *in vitro* drug-drug interaction potential. Based on the *in vitro* data, nifurtimox, M-6, and M-4 are not predicted to be inhibitors or inducers of CYP enzymes during the therapeutic use of nifurtimox. Furthermore, nifurtimox and the two metabolites are not inhibitors of BCRP, P-gp, MATE1, MATE2K, OATP1B1, OATP1B3, OAT1, OAT3, or OCT2 at clinically relevant concentrations. The only interaction of note was the inhibitory potential of M-4 towards OATP1B3. However, the IC_50_ of 10 µM predicts that this would not be of physiological relevance under normal dosing regimens.

The strengths of this investigation overall are the identification and quantification of prominent nifurtimox metabolites in patients with Chagas disease and the identification of major routes of excretion of nifurtimox and its metabolites using standard HPLC-LSC analysis in combination with contemporary HRMS. Based solely on quantitative HRMS analysis, a high proportion of the nifurtimox dose administered in humans (approximately 27% and 44% in fasted and fed patients, respectively) was found in urine by quantification of six prominent metabolites. These proportions are most likely underestimated, as many additional drug-related products were observed but not quantified in human urine due to the lack of reference standards. These findings underpin the previously observed effect of food on orally dosed nifurtimox [12] that increased the systemic exposure of fed patients (relative to fasted patients); this effect is likely to be due to a higher absorption rate, thus leading to a higher proportion of dose subsequently being excreted in the urine. However, the instability of nifurtimox in urine and feces impedes differentiation between excreted metabolites and breakdown products and compromises the interpretation of the metabolite profiles. This is because the human urine samples analyzed in this study, in which only traces of unchanged nifurtimox were found, were collected over a period of either 4 or 8 h and were not immediately frozen during collection. In addition, the human study was performed in patients with Chagas disease; the pharmacological mode of action of nifurtimox results from its reduction by *T. cruzi.* Thus, it is not possible to differentiate metabolism attributable to the disease-causing parasite from that of humans with infection. Overall, these aspects clearly hamper the identification of the exact clearance pathways and lead to the underestimation of the unchanged excretion of nifurtimox.

## CONCLUSION

The data reported in this study confirm and extend the current knowledge of nifurtimox disposition after oral administration. The unusual metabolism of nifurtimox results in a complex metabolite profile of at least 30 breakdown products, of which six metabolites (M-1 to M-6) predominate. M-6 and M-4 are the only metabolites that achieve relevant systemic exposure in humans. Based on the significant exposure of M-6 and M-4 in rats, toxicological studies on rats cover M-6- and M-4-related toxicity risks. Separate *in vitro* studies addressing the pharmacokinetic DDI potential indicated no clinical relevance. The main routes of excretion of nifurtimox and its metabolites are the renal and biliary/fecal routes, which appear to be of similar importance in rats and humans. However, the instability of nifurtimox in excreta likely resulted in the underestimation of its unchanged excretion.

## Figures and Tables

**Table 1:** Plasma PK parameters of [^14^C]-nifurtimox and metabolites^a^ after oral administration in male Wistar rats.

**Parameter**	**Total Radioactivity**	**Nifurtimox**	**M-1**	**M-2 + M-3^b^**	**M-4**	**M-5**
AUC, µg-eq*h/L	5250	1730	767	326	791	177
AUC_(0-tn)_, µg-eq*h/L	4430	1570	715	118	107	102
t_n_, h	8	5	8	3	3	5
AUC_(0-8)_, µg-eq*h/L	4430	1680	715	NC	NC	NC
%AUC_(0-8)_	100	37.9	16.1	2.66^c^	2.42^c^	2.30^c^
C_max_, µg-eq/L	1100	755	138	49.1	48.3	32.1
t_max_, h	0.5	0.5	3.0	1.5	0.5	1.5
t_½_, h	2.92	1.44	1.36	4.27	12.3	3.76

**Note:** ^a^The [^14^C]-label was missing in M-6, precluding its detection in this analysis. ^b^M-2 and M-3 could not be separated chromatographically; therefore, PK parameters were calculated for the sum of their values. ^c^Values are estimates based on individual AUC_(0-tn)_ relative to total radioactivity AUC_(0-8)_. NC was not calculated due to the high extrapolated proportion of AUC_(tn-∞)_.

**Abbreviation:** tn, time of last quantifiable concentration.

**Table 2:** Plasma PK parameters of nifurtimox and metabolites M-1 to M-6 after a single oral dose of 120 mg nifurtimox in patients with Chagas disease.

**Parameter**	**Nifurtimox**	**M-1**	**M-2**	**M-3**	**M-4**	**M-5**	**M-6**
AUC, nmol*h/L	8680	913	691	616^a^	4550^a^	256	35600
AUC, %	16.9	1.8	1.4	1.2^a^	8.9^a^	0.5	69.4
AUC_(0-24)_, nmol*h/L	8630	NC	NC	313	2350	NC	27800
AUC(t_last_-∞), %	0.6	7.6	7.8	49.2	48.4	11.7	21.8
C_max_, nmol/L	1730	113	85.4	28.4	207	30.0	2250
t_max_, h	3.0	4.0	6.0	4.0	4.0	4.0	4.0
t_½_, h	3.1	3.2	3.3	28.9^a^	27.5^a^	3.8	10.1
f_u_, %	57.6	NA	NA	NA	100	NA	100

**Note:** ^a^estimated values due to high extrapolated AUC(t_last_-∞).

**Abbreviations:** NC, not calculated; NA, not available.

## Data Availability

The data that support the findings of this study are available from the corresponding author, Simone I. Schulz, upon reasonable request.

## LIST OF ABBREVIATIONS

ADME: Absorption, Distribution, Metabolism, and Excretion
A_E_: The Amount of Radioactivity Recovered in the Excreta
AUC: Area Under the Curve
BCRP: Breast Cancer Resistance Protein
BDC: Bile Duct-Cannulated
CV: Coefficients of Variation
CYP: Cytochrome P450
DDI: Drug-Drug Interaction
GFR: Glomerular Filtration Rate
HPLC: High-Performance Liquid Chromatography
HRMS: High-Resolution Mass Spectrometry
LSC: Liquid Scintillation Counting
MATE: Multidrug and Toxin Extrusion
NA: Not Available
NC: Not Calculated
OAT: Organic Anion Transporter
OATP: Organic Anion Transporting Polypeptide
OCT: Organic Cation Transporter
P-gp: P-Glycoprotein
PK: Pharmacokinetics
