# Supplementary material for: In vivo Metabolism of Nifurtimox and the Drug-Drug Interaction Potential Including its Major Metabolites
Source: Curr Drug Metab. 2023 Oct 17;24(8):599–610. doi: 10.2174/1389200224666230817114758 (PMC10661964; doi:10.2174/1389200224666230817114758)
Supplement: Supplementary file 1 — Supplementary material is available on the publisher’s website along with the published article. [file CDM-24-599_SD1.pdf]

Supplementary Material

In vivo Metabolism of Nifurtimox and the Drug-Drug Interaction Potential Including its Major Metabolites

Simone I. Schulz*[1], Dieter Lang[1], Gabriele Schmuck[1], Michael Gerisch[1], Michaela Bairlein[1], Robert Fricke[1] and Heino Stass[1]

[1]*Bayer AG, 42096 Wuppertal, Germany*

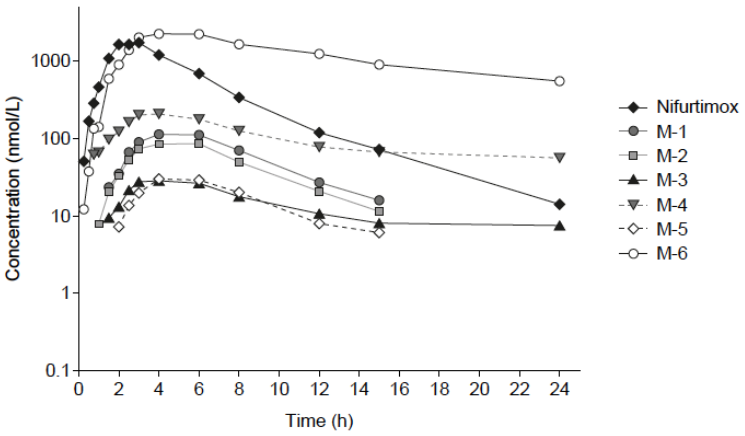

Fig. (S1). Plasma concentrations *vs* time curves of nifurtimox and metabolites M-1 to M-6 after oral administration of 120 mg nifurtimox to Chagas patients [14].

Table S1. Excretion of radioactivity after a single administration of 2.5 mg/kg [^{14}C]-nifurtimox shown as mass balance in percentage of the administered dose up to 120 h after oral dosing in intact male Wistar rats and up to 24 h after i.v. dosing in BDC male Wistar rats.

Compound	Oral dosing		i.v. dosing		
	Urine	Feces	Urine	Bile	Feces
Nifurtimox	0.117	-	0.847	0.560	-
M-1/M-5	16.6	1.54[a]	6.56	2.65	0.489[a]
M-2/M-3	4.97	3.62[c]	8.02	1.56	0.186[c]
M-4	-	-	-	0.968	-
M-10a	-	-	0.919	-	-
M-10b	-	-	1.07	-	-
M-11	3.13	-	2.27	1.09	-
M-12	0.728	-	0.934	0.289	-
M-13a	-	7.69	-	-	0.195
M-13b	-	7.03	-	-	0.184
M-14	-	2.07	-	-	0.0455
M-15	-	-	2.85	-	-
M-16	0.523	-	-	-	-
Other	22.5	21.0	26.1	10.5	1.35
Not profiled	-	0.821	-	-	-
Subtotal	48.6	43.8	49.6	17.7	2.45
Total	92.4		69.8[b]		

[a]data represents M-1 only (no M-5 was identified in feces samples)
[b]in addition, 4.45% of the dose was present in the gastrointestinal tract (GIT) including contents, 17.6% in the body excluding the GIT and 2.48% in the cage wash, yielding a total recovery of 94.2%
[c]data represents M-3 only (no M-2 was identified in feces samples)

Table S2. Plasma PK parameters of nifurtimox and metabolites M-1 to M-6 after oral administration of a single dose of 200 mg/kg nifurtimox to Wistar rats (n = 4 per group).

Parameter	Nifurtimox	M-1	M-2	M-3	M-4	M-5	M-6
Female animals	-	-	-	-	-	-	-
AUC, nmol*h/L	121000	44200	3630	3700	7410[a]	2430	96100
$AUC_{(0–24)}$, nmol*h/L	117000	36700	3220	3540	4840	1420	65800
C_{max}, nmol/L	18300	2050	447	547	568	186	3920
t_{max}, h	1.0	8.0	2.0	1.0	3.0	3.0	3.0
$t_{½}$, h	5.0	4.6	8.5	5.8	18.6[a]	25.3	13.5
Male animals	-	-	-	-	-	-	-
AUC, nmol*h/L	184000	46600	13400[a]	10100[a]	9770[a]	8020	148000
$AUC_{(0–24)}$, nmol*h/L	138000	28200	3310	3640	4780	4640	78400
C_{max}, nmol/L	10100	1390	220	255	338	328	3650
t_{max}, h	2.0	24.0	2.0	1.0	2.0	3.0	24.0
$t_{½}$, h	5.0	4.2	63.3[a]	39.6[a]	18.3[a]	8.2	10.6

[a] value estimated due to high extrapolated proportion.

Table S3. Results of *in vitro* studies of inhibition of CYP isoforms using probe substrates (A) and induction of CYP isoform expression or activity (B).

(A) inhibition

CYP Isoform	Probe Substrate	Nifurtimox			M-6			M-4		
-	-	Co- IC_{50} (µM)	Pre- IC_{50} (+ NADPH) (µM)	TDI	Co- IC_{50} (µM)	Pre- IC_{50} (+ NADPH) (µM)	TDI	Co- IC_{50} (µM)	Pre- IC_{50} (+ NADPH) (µM)	TDI
1A2	Phenacetin	>50	>50	No	>100	>100	No	>20	>20	No
2A6	Coumarin	>50	-	NA	>100	-	NA	>20	-	NA
2B6	Bupropion	>50	>50	No	>100	>100	No	>20	>20	No
2C8	Amodiaquine	>50	>50	No	>100	>100	No	>20	>20	No
2C9	Diclofenac	>50	>50	No	>100	>100	No	>20	>20	No
2C19	Mephenytoin	>50	>50	No	>100	>100	No	>20	>20	No
2D6	Dextromethorphan	>50	>50	No	>100	>100	No	>20	>20	No
2E1	Chlorzoxazone	>50	-	NA	>100	-	NA	>20	-	NA
3A4 (M)	Midazolam	>50	48–49	No	>100	>100	No	>20	>20	No
3A4 (T)	Testosterone	>50	>50	No	>100	>100	No	>20	>20	No

-, not determined; Co-, coincubation; CYP, cytochrome P450; IC_{50}, concentration required for 50 percent inhibition; NA, not applicable; NADPH, nicotinamide adenine dinucleotide phosphate; Pre-, 30-min preincubation; TDI, time-dependent inhibition.

(B) induction (mRNA and CYP activity)

CYP Isoform	Nifurtimox	M-6	M-4
1A2	No induction up to 30000 µg/L	No induction up to 90000 µg/L	No induction up to 80000 µg/L
2B6	No induction up to 30000 µg/L	No induction up to 90000 µg/L	No induction up to 80000 µg/L
2C19	No induction up to 30000 µg/L	No induction up to 90000 µg/L	No induction up to 80000 µg/L
3A4	No induction up to 30000 µg/L	No induction up to 90000 µg/L	No induction up to 80000 µg/L

CYP, cytochrome P450.

Table S4. Inhibitory effect of nifurtimox, M-6 and M-4 on selected efflux (A) and uptake (B) transporters *in vitro.*

(A) efflux transporters

Transporter	Probe Substrate	Nifurtimox IC_{50} (µM)	M-6 IC_{50} (µM)	M-4 IC_{50} (µM)
P-gp	Digoxin	>100	>200	>5[a]
P-gp	Dipyridamole	>100	>200	>20
BCRP	Topotecan	>100	>200	>20
BCRP	Fluvastatin	-	>200	>20
MATE1	Metformin	>20	>30	>5
MATE2K	Metformin	>20	>30	>5

-, not determined; BCRP, breast cancer resistance protein; P-gp, P-glycoprotein; MATE, multidrug and toxin extrusion. [a]The highest test concentration of 20 µM was not valid in this assay for technical reasons.

(B) uptake transporters

Transporter	Probe Substrate	Nifurtimox IC_{50} (µM)	M-6 IC_{50} (µM)	M-4 IC_{50} (µM)
OATP1B1	Pravastatin	>50	>50	>20
OATP1B3	Pravastatin	>50	>50	10
OAT1	*p*-Aminohippuric acid	>20	>30	>5
OAT3	Estrone-3-sulphate	>20	>30	>5
OCT2	1-Methyl-4-phenylpyridinium	>20	>30	>5

OAT, organic anion transporter; OATP, organic anion transporting polypeptide; OCT, organic cation transporters.

SUPPLEMENTARY METHODS

Determination of Inhibitory Potential of Nifurtimox, M-6 and M-4 for MATE1 and MATE2K *In Vitro*

Test System

After thawing from frozen storage, aliquots of approximately 3 x 10^6 cells of human embryonic kidney (HEK293) cell line stable overexpressing MATE1 or MATE2K were transferred to cell culture dishes containing 10 mL of DMEM-medium (supplemented with 10% FBS, 1% penicillin [10.000 U/mL]/streptomycin [10 mg/mL] and hygromycin B [150 µg/mL]) at 37°C. Transporter-transfected and vector-transfected HEK cells were grown on cell culture dishes at 37°C in a humidified 5% CO_2 atmosphere. All cell lines were confirmed free from mycoplasma by routine testing with conventional PCR. Cell cultures were split when they had grown to confluency. For passaging, cells were washed with PBS, exposed to trypsin-EDTA for 5 min at room temperature, collected by centrifugation, resuspended in culture medium and re-incubated.

MATE1 and MATE2K Transport Assays

Transporter-transfected and vector-transfected cells were prepared for use in uptake assays by harvesting and plating into 24-well-plates pre-coated with poly-D-lysine hydrobromide at a density of 2 x 10^5 cells/well and then cultured for 3 days in DMEM growth medium (high glucose, 10% FBS, 1% penicillin/streptomycin). For uptake assays, the growth medium was aspirated and each plate well was rinsed twice with HBSS supplemented with 20 mM HEPES, pH 7.4. The intracellular environment was acidified by preincubation in 30 mM NH_4Cl in buffer pH 7.4 at 37°C for 30 min. The buffer was then removed and 200 µL incubation buffer containing substrates with or without test compound or the respective probe inhibitor was added to each well. Assay plates were then incubated at 37°C for specified times. After incubation, the uptake was terminated by aspirating the reaction mixture and washing the cells three times with 0.4 mL ice-cold PBS buffer. Cells were solubilized with 0.6 mL of 1 M NaOH overnight. The contents of each well (0.6 mL) was transferred into a scintillation vial and the [^{14}C]-content was measured after addition of 2.5 mL scintillation solvent (Roti® eco plus, Carl Roth) in a liquid scintillation counter.

To assay potential MATE1 inhibition, the uptake of the [^{14}C]-labeled probe substrate ([^{14}C]-metformin) and non-labeled metformin was performed at a final concentration of 20 µM. To assay potential MATE2K inhibition, the uptake of the [^{14}C]-labeled and unlabeled substrates was performed at a final concentration of 90 µM. In both assays, the inhibitory effect of nifurtimox, M-6 and M-4 was determined by adding two concentrations to the [^{14}C]-labeled probe substrate as follows: nifurtimox (2 µM and 20 µM), M-6 (3 µM and 30 µM) and M-4 (0.5 µM and 5 µM). Inhibition of MATE1 and MATE2K-mediated [^{14}C]-metformin uptake by cimetidine (50 µM) was performed as a positive control in parallel. Uptake was terminated after incubation for 1 min. The uptake of the [^{14}C]-labeled compound was quantified by liquid scintillation counting. The absolute amount of the substrate uptake was calculated for the incubation time and related to the determined amounts of cellular protein.
